# Family-Based Study Reveals *PDE11A/PDE11A-AS1* Variants in Testicular Germ Cell Tumor Predisposition

**DOI:** 10.3390/ijms27125261

**Published:** 2026-06-10

**Authors:** Luiza Côrtes, Ana Beatriz Rodrigues, Sara Martoreli Silveira, Julieta M. Ramírez-Mejía, Carine Spenassatto Dreyer, Mads M. Aagaard, Geysson Javier Fernandez, Ademar Lopes, José Carlos S. Trindade Filho, Silvia Regina Rogatto

**Affiliations:** 1 Botucatu Medical School Hospital (HCFMB), São Paulo State University—UNESP, Botucatu 18618-687, SP, Brazil; luiza.cortes@unesp.br (L.C.); ana-beatriz.rodrigues@unesp.br (A.B.R.); carine.dreyer@unesp.br (C.S.D.); jc.trindade@unesp.br (J.C.S.T.F.); 2 Graduation Program in Genetics, Biosciences Institute, São Paulo State University—UNESP, Botucatu 18618-689, SP, Brazil; 3 Teaching Department, Universidade Nove de Julho (UNINOVE), São Paulo 01504-001, SP, Brazil; s.m.binato@uni9.pro.br; 4 Laboratorio de Biología y Control de Enfermedades Infecciosas (BCEI), Sede de Investigación Universitaria (SIU), Facultad de Ciencias Exactas y Naturales, Instituto de Biología, Universidad de Antioquia, Medellín 050010, Colombia; jmramirez2@eafit.edu.co (J.M.R.-M.); geysson.fernandez@udea.edu.co (G.J.F.); 5 Clinical Genetics Department, University Hospital of Southern Denmark, 7100 Vejle, Denmark; mads.jorgensen@rsyd.dk; 6 Department of Pelvic Surgery, A.C. Camargo Cancer Center, São Paulo 01509-001, SP, Brazil; ademarlopes@uol.com.br; 7 Institute of Regional Health Research, University of Southern Denmark, 5230 Odense, Denmark

**Keywords:** testicular cancer, germ cell tumor, whole exome sequencing, *PDE11A*, *PDE11A-AS1*

## Abstract

Testicular germ cell tumor (TGCT) is a common tumor type in young men. Family history of TGCT and its presence in twins support the involvement of inherited genetic factors. Germline exome sequencing was performed on monozygotic twins with TGCT and their parents. The twins presented compound heterozygous variants in *PDE11A* (rs776984134 and rs17400325) inherited from each parent. The rs776984134 variant disrupts the canonical splice acceptor site, leading to aberrant splicing and a frameshift predicted to affect protein structure. The rs17400325 missense variant, located in the catalytic domain, reduces hydrogen bonding capacity and may impair protein stability. Both variants map to a genomic region overlapping the antisense lncRNA *PDE11A-AS1*. In silico transcript-level analysis predicted multiple energetically favorable RNA–RNA interactions between *PDE11A* and *PDE11A-AS1* transcripts, with rs17400325 located within predicted hybridization regions of several isoforms. These results suggest a potential impact on *PDE11A–PDE11A-AS1* pairing and post-transcriptional regulation. Additional variants in *MSH6* and *CTU2* were also identified and may act as potential modifiers of disease susceptibility, consistent with a multigenic contribution to TGCT risk. These findings support a contributory role for the *PDE11A* locus in TGCT predisposition and underscore the biological relevance of overlapping sense–antisense genomic regions in hereditary cancer studies.

## 1. Introduction

Testicular germ cell tumor (TGCT) is the most prevalent malignant neoplasm among young men, with the highest incidence occurring between 15 and 40 years of age, representing approximately 1% of all cancer cases diagnosed worldwide [1,2]. The main risk factors for TGCT include cryptorchidism (>5 times the risk of developing TGCT), hypospadias, family history of the disease, tumors in the contralateral testicle, and testicular dysgenesis syndrome [3,4]. Patients with a family history of TGCT (seminomas or non-seminomas) in first-degree relatives have 5,06 fold increased risk of developing the disease [5]. In 97,402 first-degree relatives of 21,254 testicular cancer patients, Kharazmi et al. [6] reported that the lifetime cumulative risk of testicular cancer in brothers was 2.3%, and for sons, it was 1.2%. The risk increased to 10–11% when two or more family members were affected by the disease. In twins, the lifetime risk of developing TGCT ranged from 9% to 74%, depending on the age at diagnosis [6].

Treatment of germ cell tumors is determined by histological type, stage, and serum tumor marker status (human chorionic gonadotropin, alpha-fetoprotein, lactate dehydrogenase) and may involve surgery, chemotherapy, and radiotherapy [7]. Cisplatin-based chemotherapy increased five-year survival rates to 95% [8]. Advancements in the diagnosis and treatment of testicular cancer have significantly improved patient survival, with cure rates exceeding 90%, even in metastatic cases [9]. TGCT patients have a 5-year survival rate of 99%, 92%, and 85% for stages I, II, and III, respectively [2].

Pyle et al. [10] described pathogenic candidate variants and potential therapeutic targets (such as *CFTR*, *PIM1*, and *CRBN*) in a large familial TGCT study using exome sequencing [10]. A complex polygenic model with significant genetic variability was suggested to explain the inheritance of TGCT [10]. Single-nucleotide polymorphisms (SNPs) have been associated with genetic familial risk of TGCT development. Candidate genes include those involved in germ cell differentiation, ciliary-microtubule function, and the *KIT/KITLG* pathway, such as rare deleterious variants in *PDE11A* (i.e., *p.R545X* and p.K568R) [11,12,13]. Variants in the *PIM1, ZP4, NIN*, and *QRSL1* genes, which are involved in chromosomal segregation and cell cycle regulation, have also been described [10]. Although with moderate penetrance, pathogenic/likely pathogenic (P/LP) *CHEK2* variants have been reported, supporting its role as a predisposition gene in TGCT [10,14,15]. Recently, Ugalde-Morales et al. [16] described a transcriptome-wide association study that identified 165 TGCT-associated genes. The authors selected 46 candidate genes (GWAS-inflated signals, correlations between neighboring genes, and evidence of colocalization), of which 23 overlapped with 22 genome-wide association studies loci, including seven not previously implicated in TGCT risk. They also confirmed increased protein expression of ARID3B and GINM1, suggesting their roles in genetic predisposition to TGCT [16]. Predisposition may result from a combination of moderate- and low-risk variants, influenced by testicular structure or alterations in temperature, favoring fetal testicular germ cell proliferation or delayed maturation [14].

In this study, we conducted germline exome sequencing on TGCT monozygotic twins and their parents, followed by Sanger sequencing to validate selected variants. We then performed exploratory in silico functional predictions of these variants. Our findings revealed a compound heterozygous genotype in *PDE11A/PDE11A-AS1*, supporting its role as a candidate genetic predisposition locus for TGCT.

## 2. Results

**Figure 1:**
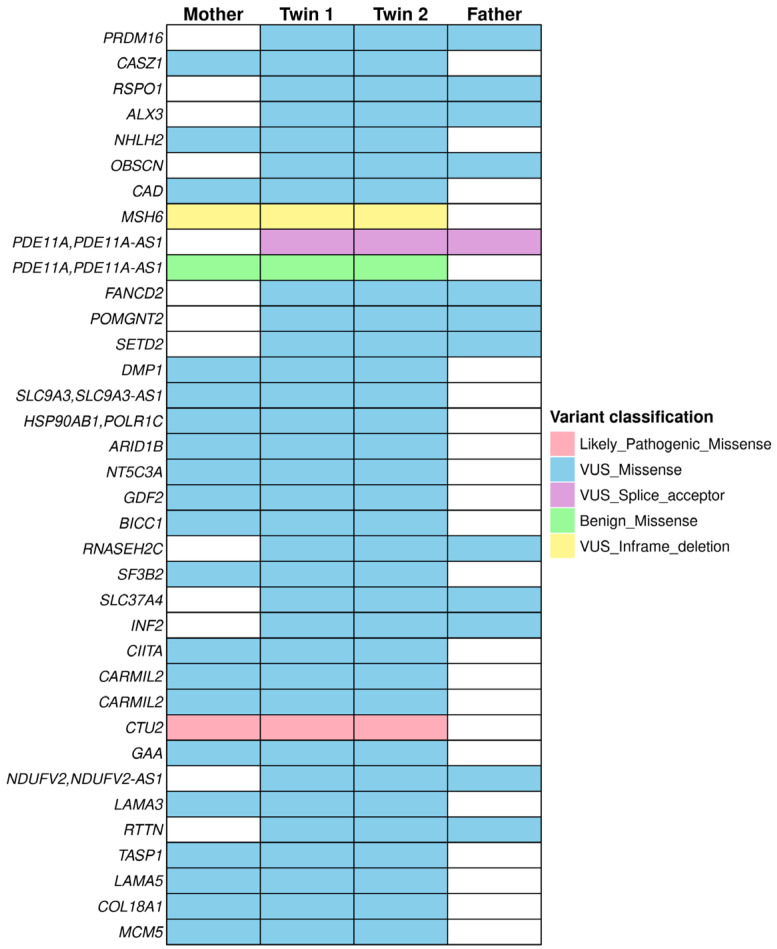
OncoPrint illustrating the distribution and inheritance patterns of germline variants in twins with testicular germ cell tumor and their parents.

We identified 32 top-prioritized genes with a family-based context (Table 1 and Figure 1). Variants in genes with weak association with testicular cancer were detected, as indicated by the sample Phorank gene score (Table 1), including *OBSCN, CAD, DMP1, NT5C3A, SF3B2, CIITA, NDUFV2,* and *COL18A1.* Among the potentially damaging variants, only rs147948789, which mapped to *CTU2*, was classified as likely pathogenic; the remaining were VUS, except rs17400325, which mapped to *PDE11A* (benign ACMG/not classified ClinVar).

**Figure 2:**
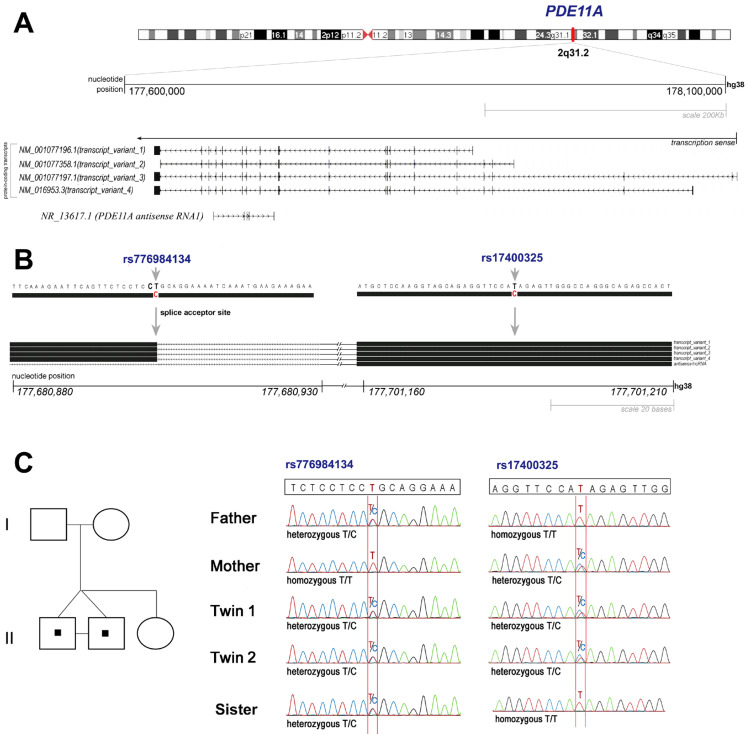
Genetic variants of *PDE11A* identified in a family of twins with testicular germ cell tumors. (**A**) Schematic representation of the *PDE11A* gene mapped at 2q31.2. (**B**) variants localization of rs776984134 (splice acceptor site—intron 12 and exon 13 junction) and rs17400325 (exon 17) identified in the family members; (**C**) Pedigree representative of the family (left) and electropherograms (right) showing the segregation of the variants: rs776984134 detected in the father, sister (II-3), and twins; rs17400325 detected in the mother and twins.

The twins presented a *PDE11A* compound heterozygous genotype (rs776984134; c.2346-2A>G and rs17400325; c.2180A>G). Their father carried the rs776984134 (splice acceptor & intron variant), and their mother the missense variant rs17400325. The rs776984134 and rs17400325 variants map to intron 12 and exon 17 of the *PDE11A* gene, respectively (Figure 2A). Both variants map within the catalytic domain, which spans amino acids 663–839, suggesting potential functional consequences for enzymatic activity. Based on these findings, we elected *PDE11A* as a strong candidate gene for a TGCT predisposition in this family.

**Figure 3:**
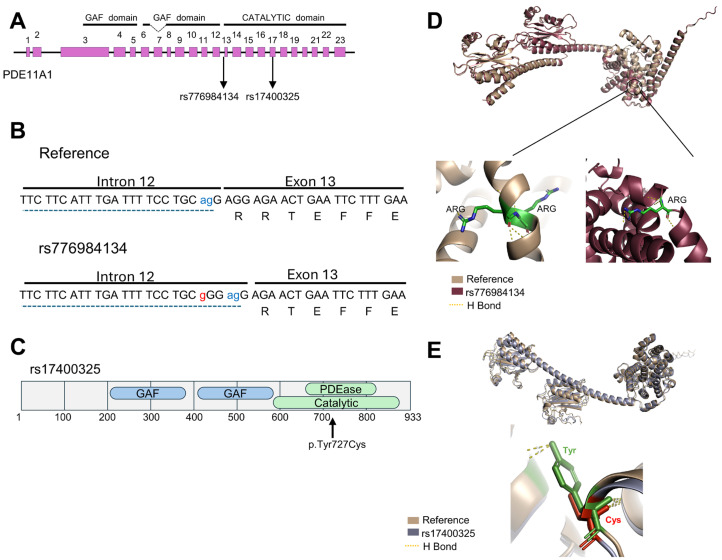
Functional annotation of *PDE11A* variants. (**A**) The rs776984134 and rs17400325 variants map to the catalytic domain of the PDE11A protein. The rs776984134 variant is positioned within intron 12, while the rs17400325 variant is in exon 17. (**B**) The diagram illustrates the junction between intron 12 and exon 13, showing the rs776984134 variant (red), the AG splice site (blue), which is recognized during splicing, and the corresponding amino acid sequence encoded by exon 13. (**C**) Domain map of the PDE11A protein highlighting that the rs17400325 variant results in a p.Tyr727Cys substitution within the catalytic domain. The GAF domains (blue) are located at amino acids 217–380 and 402–568, while the HD/PDEase catalytic domain (green) spans amino acids 663–839. Predicted three-dimensional structures of reference and mutant PDE11A proteins. Structural comparisons between the PDE11A wild type and the mutant proteins carrying the rs776984134 (**D**) and rs17400325 (**E**) prediction analysis revealed that both variants are predicted to reduce hydrogen bonding, leading to destabilization of the alpha-helix structure.

The rs776984134 variant disrupts the canonical AG splice site at the intron 12 and exon 13 junction, as shown in the splice site diagram (Figure 3B). This disruption redirects splicing to an alternative AG site, resulting in a frameshift that alters the protein sequence downstream of exon 13, including the loss of the arginine residue. Predicted three-dimensional structures of the wild-type and mutant PDE11A proteins revealed that the loss of this arginine reduces hydrogen bonding in the affected region, potentially affecting the stabilization between adjacent alpha helices near the catalytic domain (Figure 3D). These findings suggest that the rs776984134 variant, located near the splice acceptor site, may affect *PDE11A* function by impairing protein stability and structural integrity.

The *PDE11A* second variant, rs17400325, was categorized as benign according to ACMG guidelines. However, it introduces a p.Tyr727Cys substitution within the HD/PDEase catalytic domain, a region identified as critical for the enzymatic function of the PDE11A protein (Figure 3C). Functional impact analysis using PolyPhen-2 classified the variant as potentially damaging with a high predictive score of 0.995, sensitivity of 0.45, and specificity of 0.96 (Supplementary Figure S2A), indicating a substantial likelihood of functional disruption. Structural analysis of three-dimensional models of the wild-type and mutant PDE11A proteins revealed that substituting tyrosine with cysteine reduces hydrogen-bonding capacity (Figure 3E). Tyrosine, with its hydroxyl group, may form stronger and more numerous hydrogen bonds, which are crucial for stabilizing protein structures and facilitating interactions. In contrast, cysteine’s thiol group is less effective in hydrogen bonding, potentially destabilizing the catalytic domain and impairing enzymatic function.

The two *PDE11A* variants identified are located within a genomic region overlapping the antisense lncRNA *PDE11A-AS1,* which is transcribed from the same locus (Figure S2B), suggesting that they influence the structure and function of the lncRNA. To better investigate this finding, we analyzed the secondary structure of *PDE11A-AS1* using CentroidFold. (http://www.ncrna.org/centroidfold/, accessed on 5 March 2025). We observed subtle structural differences (Figure S2C), which may indicate a functional impact on the lncRNA *PDE11A-AS1*.

Annotation of rs17400325 using the Variant Effect Predictor (VEP) showed that the variant maps to multiple PDE11A protein-coding transcripts, where it is consistently classified as a missense variant with a predicted moderate impact. In the antisense *PDE11A-AS1*, the variant overlaps exon 2 of transcript ENST00000412133, whereas it maps to intronic regions in the remaining annotated transcripts (Table S1).

To investigate potential regulatory RNA–RNA interactions, all pairwise combinations of sense *PDE11A* and antisense *PDE11A-AS1* transcripts were analyzed, yielding 60 transcript pairs. Among these, a subset of *PDE11A* transcripts (ENST00000286063, ENST00000358450, ENST00000389683, ENST00000409504, ENST00000433879, and ENST00000497003) exhibited highly favorable interactions with the *PDE11A-AS1* transcript ENST00000412133. IntaRNA predicted strongly negative hybridization energies for these pairs, reaching −148.39 kcal/mol, consistent with the formation of energetically stable RNA duplexes (Table S2).

Collectively, these findings delineate a transcript-level interaction landscape between *PDE11A* and the *PDE11A-AS1*, characterized by recurrent duplex formation predicted to be energetically favorable (Figure S5). The rs17400325 variant lies within predicted RNA–RNA hybridization regions across multiple transcript isoforms, suggesting a potential impact on *PDE11A–PDE11A-AS1* pairing and post-transcriptional regulation.

The sequence of *PDE11A-AS1* transcript NR.136171.1 (fragment 776–827) was analyzed using catRAPID omics (https://service.tartaglialab.com/page/catrapid_group, accessed on 21 January 2026), which is predicted to interact with a diverse set of proteins (Table S3). Functional enrichment analysis of these predicted interactors revealed enrichment for RNA-associated activities, including RNA binding, nucleic acid binding, and RNA helicase activity. Additional enriched terms indicated ATP-dependent molecular functions and broader nucleotide-hydrolyzing processes. Biological Process and Cellular Component categories further supported the involvement of these proteins in spliceosome assembly, nuclear export (GO:0051168), and major ribonucleoprotein complexes (Figure S3). Together, these predictions suggest that *PDE11A-AS1* may act as a multifunctional lncRNA, potentially modulating RNA metabolism, post-transcriptional regulation, and nuclear RNA–protein complex formation through selective interactions with RNA-binding proteins.

Given the critical role of lncRNAs in the competing endogenous RNA (ceRNA) hypothesis, we examined the variant’s potential to alter miRNA binding using the database LncRNASNP2. The analysis revealed that the rs17400325 variant increased targeting of miR-7114-5p, miR-6833-5p, and miR-3664-5p, while reducing binding by miR-618. Although the role of the lncRNA *PDE11A-AS1* remains unknown, these findings suggest that the rs17400325 variant may affect the regulatory process involving miRNAs.

In addition to *PDE11A, MSH6* was identified as a potential candidate gene, ranking among the highest-scoring genes in the Sample PhenoRank analysis. A heterozygous in-frame deletion of *MSH6* (rs587782858; c.2643_2645del; p.Lys852del) was identified in both twins and their mother. According to the MANE Select transcript (NM_000179.3/ENST00000234420.11), the variant is mapped on exon 4 and results in the deletion of a single lysine residue at position 852 of the MSH6 protein, potentially altering its structure or function by disrupting a conserved amino acid. Domain annotation performed with InterPro mapped the variant to the DNA repair protein MutS, Domain III (IPR036187; amino acids 736–1068), a region implicated in signal transduction between the ATPase and DNA-binding domains of *MSH6*. The p.Lys852 in MSH6 was further examined using AlphaFold-based three-dimensional structural modeling. Models spanning residues 608–1082 were generated for both wild-type and rs587782858 variant proteins. Structural alignment indicated that the overall protein fold was preserved (RMSD = 0.687 Å), suggesting no major conformational changes. Local differences were observed at the deletion site, with analysis of Cα–Cα distances revealing a 28.8% reduction in backbone length in the variant compared with the wild-type (from 5.428 Å to 3.865 Å), suggesting a local contraction of the polypeptide chain.

Variants in *PDE11A, MSH6,* and *CTU2* were selected for validation based on prioritization criteria that considered predicted functional impact and relevance in this family. For *PDE11A,* Sanger sequencing confirmed these variants in all family members, including the twins’ sister (evaluated exclusively by Sanger sequencing), who harbors the rs776984134 variant (Figure 2C). Variants in *MSH6* and *CTU2* were also confirmed in the twins and their mother (Figure S6).

To provide additional biological context for the candidate genes identified in this study, we evaluated public transcriptomic datasets from GTEx and TCGA-TGCT. *PDE11A* showed high expression in normal testicular tissues and reduced expression in TGCT samples, whereas *MSH6* and *CTU2* exhibited increased expression in tumor samples compared to normal testis (Figure S7A).

In addition, we performed a systems biology analysis focusing on proteins functionally connected to PDE11A. The resulting interaction network included proteins involved in nucleotide metabolism and cyclic nucleotide signaling, such as GUK1, GMPR, ITPA, DCK, AK3, APRT, ADK, ENPP1, ENPP3, and PRKAR1A (Figure S7B). Functional enrichment analysis revealed significant associations with biological processes related to nucleoside triphosphate metabolism, pyrimidine nucleotide metabolism, purine ribonucleotide metabolism, nucleoside phosphate metabolism, and regulation of cAMP-dependent protein kinase signaling (Figure S7C).

## 3. Discussion

We examined the spectrum of germline variants in one family with twins affected by testicular germ cell cancer, using whole-exome sequencing. Rare variants were retained in a list of 32 top-prioritized genes. Among the detected genes, the *PDE11A,* which is highly expressed in the testis, was a strong candidate for hereditary TGCT predisposition. *PDE11A* encodes a dual-specificity phosphodiesterase that hydrolyzes cAMP and cGMP, modulating cellular signaling pathways involved in cell proliferation, differentiation, metabolism, and oncogenesis [17,18]. The gene encodes four isoforms (*PDE11A1*–*PDE11A4*) with distinct amino-terminal sequences. The isoform 4 is highly expressed in testicular tissue, the only tissue known to contain all four isoforms [17]. Single-nucleotide variants in this gene have been associated with testicular cancer in patients with a family history of this tumor type [19,20]. The analysis of public transcriptomic datasets revealed reduced *PDE11A* expression in TGCT samples compared with normal testicular tissues (Figure S7A). In agreement, Horvath et al. [19] reported that inactivating *PDE11A* variants may contribute to susceptibility to familial and bilateral TGCT and are associated with lower *PDE11A* expression in affected tumor tissues. Similar patterns of reduced *PDE11A* expression have also been described in prostate cancer by Faucz et al. [21], including lower protein levels in tumor samples compared with adjacent normal tissues.

Polymorphisms in *PDE11A* have been associated with increased TGCT risk and altered sperm counts, suggesting a potential association between *PDE11A* variants and testicular function [22]. Previous studies described *PDE11A* alterations to impaired sperm parameters, hormonal dysregulation, and increased susceptibility to endocrine and testicular tumors [19,23,24]. Furthermore, phosphodiesterase (PDE) inhibition may influence hypoxia-related mechanisms of chemoresistance in prostate cancer models [25]. Although direct evidence for *PDE11A* involvement in cisplatin response in TGCT is currently lacking, these findings suggest that cyclic nucleotide signaling pathways may also influence tumor progression and treatment response in TGCT [25].

Genome-wide association studies have also linked *PDE11A* variants to various tumor types, including adrenal tumors and glioblastomas [22,26]. Pathak et al. [11] reported 55 germline *PDE11A* variants (20 missense, 4 splice-site, 2 nonsense, 7 synonymous, and 22 intronic) in a large cohort of TGCT patients (259 familial, sporadic, bilateral tumors). Unlike moderate-penetrance susceptibility genes such as *CHEK2*, which have been associated with familial TGCT [10,15], the contribution of *PDE11A* variants remains less clearly defined and may involve distinct biological mechanisms related to cyclic nucleotide signaling and transcript-level regulation. TGCT susceptibility has also been linked to alterations in pathways involving *KIT/KITLG* signaling and DNA repair mechanisms, supporting the concept that multiple low- and moderate-risk variants may collectively contribute to TGCT predisposition [27,28]. Although the current evidence remains insufficient for direct clinical application, the identification of rare *PDE11A* variants in familial settings may contribute to genetic risk assessment strategies in high-risk TGCT families, particularly if interpreted alongside additional susceptibility factors. Further validation in a large cohort of familial TGCT cases is necessary to determine the penetrance, predictive value, and potential utility of *PDE11A* evaluation in hereditary cancer risk investigations.

Herein, we identified compound heterozygous variants of the *PDE11A* gene in the monozygotic twins diagnosed with TGCT. The *PDE11A* variants, rs776984134 (c.2346-2A>G) and rs17400325 (c.2180A>G), were confirmed by Sanger sequencing in all family members (parents, twins, and the twins’ sister). The presence of rs776984134 in unaffected family members indicates that this splice-site variant alone may not be sufficient to promote disease development. Notably, neither the father nor the sister carrying this alteration has developed tumor types previously associated with *PDE11A.* Thus, disease development in this family may be related to a critical reduction in *PDE11A* expression, potentially acting in concert with additional genetic alterations identified in these patients, supporting a broader multigenic susceptibility model. Prediction analysis suggested that these variants may affect the catalytic domain of the PDE11A protein. The rs776984134 variant alters RNA splicing, resulting in the loss of critical residues, such as arginine, that are essential for the stability of interactions between adjacent alpha helices near the catalytic domain. Although rs17400325 is classified by ACMG as benign (gnomAD Variant frequency of 0.0369668), this variant causes the p.Tyr727Cys substitution, which weakens hydrogen bond formation in the catalytic domain and may reduce the protein’s stability and enzymatic activity. By affecting cAMP signaling, *PDE11A* variants may alter the tumor microenvironment, potentially driving proliferation, tumor progression, and changes in therapeutic response [11,12].

The identification of *PDE11A* compound heterozygosity highlights a complex genomic context with overlapping sense–antisense transcription. As previously proposed [29,30], the hereditary predisposition may involve RNA duplex formation and co-transcriptional regulation mediated by natural antisense transcripts (NATs). NATs result from bidirectional transcription and regulate gene expression through RNA–RNA interactions and post-transcriptional mechanisms. Mechanisms involving double-stranded RNA intermediates (dsRNA) are more prominent in germ cells than in somatic tissues [30]. The variant rs17400325 may be relevant due to its localization within an exonic region overlapping the antisense lncRNA *PDE11A-AS1*.

The highly negative hybridization energies observed in our analyses (~−148.39 kcal/mol) indicated energetically favorable RNA–RNA pairing between *PDE11A* and *PDE11A-AS1*, with rs17400325 located within predicted hybridization regions shared by multiple transcript pairs. Sense–antisense duplex formation has been implicated in the regulation of mRNA stability and degradation, as well as in alternative splicing within overlapping genomic regions [29,30,31]. The presence of rs17400325 within the predicted hybridization region suggests that this substitution may alter duplex stability or RNA–protein interactions and consequently affect post-transcriptional regulation at the *PDE11A* locus.

To our knowledge, *PDE11A-AS1* has not yet been functionally characterized, and no studies have investigated antisense-mediated regulation at the *PDE11A* locus. Predicted interactors of *PDE11A-AS1* are enriched for RNA-binding proteins and spliceosomal components, which aligns with previous reports indicating that natural antisense transcripts frequently participate in RNA metabolism and splicing regulation [29,30,31].

Predicted alterations in miRNA binding associated with rs17400325 provide an additional potential regulatory mechanism. Antisense transcripts can modulate gene expression by masking miRNA binding sites or acting as competing endogenous RNAs (ceRNAs) [29,31]. In line with these mechanisms, the predicted increase in binding sites for miR-7114-5p and miR-6833-5p positions the antisense transcript as an anomalous miRNA sponge [29,32]. These predicted alterations may influence ceRNA-related regulatory mechanisms at the *PDE11A* locus [29,30]. Although the functional relevance of the predicted changes in miRNA levels requires experimental validation, these findings suggest that rs17400325 may exert regulatory effects beyond its structural impact on *PDE11A*.

The pathogenesis of TGCTs in the twins may involve a deleterious synergistic effect. The paternal variant rs776984134 disrupts canonical splicing and compromises protein structure, while the maternal variant may impair the antisense regulatory system. Collectively, these variants may contribute to *PDE11A* loss of function and reduced expression, analogous to splicing and epigenetic defects involving natural antisense transcripts described in other malignancies by Dallosso et al. [33].

The variant of uncertain significance rs587782858 (*MSH6*; c.2643_2645del; p.Lys852del) was also identified in the twins and their mother. *MSH6* (MutS homolog 6) is essential for maintaining genomic stability through the mismatch repair system (MMR). In testicular germ cell tumors, MMR system integrity is critical in the germinal epithelium, as elevated rates of cellular proliferation and meiotic events require rigorous genomic surveillance [16]. The importance of *MSH6* in TGCT is supported by its high expression in primordial germ cells, suggesting a potential protective role during gonadal development [16]. Consistent with these observations, TCGA and GTEx analyses also showed increased *MSH6* expression in tumor samples compared with normal testicular tissues (Figure S7A). A large-scale transcriptome-wide association (TWAS) study identified *MSH6* as a candidate gene associated with TGCT susceptibility, based on genetically predicted gene expression and colocalization with GWAS [16]. However, TWAS findings indicate associations at the level of gene regulation and do not establish the functional impact of specific rare germline variants.

The potential structural consequences of the *MSH6* p.Lys852del variant (AlphaFold-based modeling) indicated that it preserves the overall protein fold but introduces local structural perturbations within Domain III, including reduced backbone distances and changes in model confidence (pLDDT) (Figure S4). Given that Domain III coordinates conformational transitions between the DNA-binding and ATPase domains [34,35], these findings may have implications for protein dynamics, although no direct functional impact can be concluded from in silico analysis. There is no consistent epidemiological evidence linking heterozygous *MSH6* variants to a significantly increased risk of TGCT. The variant was identified as heterozygous and was found in an unaffected family member, suggesting that this alteration alone is unlikely to be sufficient to drive disease development. Therefore, the p.Lys852del variant is more likely to act as a potential modifier within a broader multigenic model of TGCT susceptibility. Its coexistence with other variants identified in this family, including those in *PDE11A*, may contribute to disease risk through combined effects rather than a single-gene mechanism.

A likely pathogenic variant, *CTU2* rs147948789 (c.188T>C; p.Leu63Pro), was identified in both the mother and both monozygotic twins. *CTU2* (cytosolic thiouridylase subunit 2) is involved in post-transcriptional modification of cytosolic tRNAs [36,37], thereby contributing to translational fidelity, optimizing the decoding of specific codons, and maintaining proteome integrity under cellular stress conditions [37,38]. According to Wang et al. [37], *CTU2* is a potential diagnostic and therapeutic target across various cancer types. The authors demonstrated that *CTU2* expression increases with clinical stage in multiple tumor types, including TGCT. Expression analysis of the TCGA and GTEx datasets shows that *CTU2* is significantly overexpressed in tumor samples compared with normal testicular tissues (Figure S7A). In vitro experiments further confirmed that elevated *CTU2* expression enhances cellular proliferation and migration, supporting a potential role for *CTU2* in tumor progression [37].

The same rare germline variant was previously reported by Shaheen et al. [36] as a causative driver of DREAM-PL syndrome. This multisystemic recessive disorder is characterized by ambiguous genitalia in affected males. Functional assays demonstrated that substitution of leucine at position 63 with proline severely compromises *CTU2* enzymatic activity, resulting in defective 2-thiolation of the wobble uridine (U34) in tRNA-Glu, tRNA-Lys, and tRNA-Gln [36]. While there is currently no evidence that heterozygous *CTU2* variants independently drive TGCT susceptibility, the functional relevance of the p.Leu63Pro substitution and the role of CTU2 in translational regulation suggest that this variant may function as a modifier within a broader multigenic susceptibility context. The clinical discordance between the unaffected mother and the affected twins, combined with the rarity of this variant, underscores the need for additional functional studies to clarify the role of *CTU2* variants in male germ cell tumorigenesis.

Although our findings support *PDE11A/PDE11A-AS1* as a candidate locus in familial TGCT and highlight how overlapping sense–antisense genomic architecture can contribute to variant interpretation, our study has limitations. First, we evaluated only a single family, and monozygotic twins do not offer independent genetic replication. Second, the main mechanistic claims rely on computational predictions without RNA or protein validation (formalin-fixed, paraffin-embedded tumor tissue was exhausted, and no frozen tumor tissue was available). Third, expression data were not available to test the antisense regulatory model. In addition, the absence of experimentally validated *PDE11A* and *PDE11A-AS1* interaction networks currently limits a broader systems biology interpretation of this locus. Future work should include transcript-level validation of the splicing defect, assessment of PDE11A enzymatic activity, and analysis of *PDE11A-AS1* expression in tumor tissues of additional cases of familial TGCT.

## 4. Materials and Methods

### 4.1. Patients

Monozygotic twins diagnosed with testicular germ cell tumors (non-seminomatous mixed germ cell tumor and seminoma) were diagnosed, treated, and followed (10 years) at A.C.Camargo Cancer Center, São Paulo, Brazil. The twin 1 at age 21 years old presented a mixed germ cell tumor (right testicle) (pT2NxMx) containing components of embryonal carcinoma (95%) and mature teratoma (5%). A mature teratoma (clinical stage IA) was diagnosed at age 24, followed by left orchiectomy. Twin-2, at age 22 years old, developed a seminoma (pT1NxMx) (right testicle) with absence of vascular and lymphatic invasion. After 15 months was diagnosed with a classic seminoma, without any other germ cell component. After 10 years of follow-up, the patients remained free of disease. The parents have no personal or family history of cancer. The Human Research Ethics Committee approved the study (CEP# 2301/16, CAAE 61895016.0.0000.5432). The family members were advised of the procedures and provided written informed consent.

### 4.2. Whole Exome Sequencing (WES) and Sanger Sequencing

WES was performed in twins and their parents. Genomic DNA from blood samples was extracted using Qiacube DNA Blood kit (Qiagen, Valencia, CA, USA), following the manufacturer’s instructions. DNA library preparation and WES were performed using the Exome Nextera Enrichment kit and sequenced on an Illumina HiSeq 2500 (San Diego, CA, USA), generating a paired-end of 2 × 100 bp reads, as previously described [39,40]. The reads were aligned using Burrows–Wheeler Alignment (BWA-MEM), recalibrated, and realigned with the Genome Analysis Toolkit (GATK) v.4.6.2.0. The alignment statistics were performed with Sequence Alignment/Map (SAMtools) v.1.22, Picard v.3.4.0, and Binary Alignment Map (BAMtools) v.2.5.3 according to the human reference assembly hg38/GRCh38, as described in Cury et al. [40]. Variant calling and merging were performed using GATK HaplotypeCaller v.4.2.x. Germline called variants were selected using VarSeq™ version 3.0 (Golden Helix, Inc., Bozeman, MT, www.goldenhelix.com). Quality control criteria included a minimum read depth of ≥10 and a genotype quality score > 20. Variants were retained if these thresholds were met in at least one twin. Population frequency filtering was initially conducted with gnomAD v4.1, retaining variants with an allele frequency < 0.05 or variants with missing frequency data, followed by additional filtering using the AbraOM database (https://abraom.ib.usp.br/, accessed on 25 November 2025) with the same frequency threshold. Pathogenic (P) and likely pathogenic (LP) variants were prioritized according to the American College of Medical Genetics and Genomics (ACMG) guidelines and ClinVar classification. Variants of uncertain significance (VUS) were further evaluated and retained if predicted to be potentially damaging by at least two independents in silico functional prediction algorithms. Functional impact was assessed using SIFT (Sorting Intolerant From Tolerant), MutationTaster, MutationAssessor, Functional Analysis through Hidden Markov Models (FATHMM), or a Combined Annotation Dependent Depletion (CADD) score > 3. Only loss-of-function or missense variants annotated in RefSeq genes (release 110) were considered. These variants were evaluated in a family-based context for consistency with plausible inheritance models, including de novo variants, X-linked recessive inheritance from the mother in male offspring, compound heterozygosity, or autosomal dominant inheritance. Phenotype-driven gene prioritization was performed using the Sample PhenoRank gene-ranking algorithm. Human Phenotype Ontology (HPO) terms related to testicular germ cell tumor were applied (HP:0010785, HP:0010786, HP:0007379, HP:0010788, HP:0100617, HP:0100616, HP:0100005, HP:0000030, HP:0025451, and HP:0034380). Variants were prioritized when associated genes had a PhenoRank score > 0.3, integrating phenotype similarity, inheritance model, and predicted variant effect. Figure S1 summarizes the WES analysis pipeline used in our study.

Sanger sequencing was performed on family members to confirm two *PDE11A* variants identified by WES: rs776984134 (Forward: 5′-TTGAAATGGTGATGCTCCAA-3′; Reverse: 5′-GCCAAAAGCAAGAAAAGCAG-3′) and rs17400325 (Forward: 5′-CGATGGTTTTTGATGTTCCA-3′; Reverse: 5′-TGCTTTAGCCTCATGATTTCAA-3′). In addition, this method was employed to confirm the rs147948789 variant in *CTU2* (Forward: 5′-CTGCCCTCGTTTGCTGGATG-3′; Reverse: 5′-ATCGAGTCTGTCCTCAAAGGC-3′) and the rs587782858 variant in *MSH6* (Forward: 5′-GCTTTGTGCCCCACTCTGTA-3′; Reverse: 5′-GCCAATTCTGTTGCGCTGTT-3′). Chromatograms were generated using Mutation Surveyor software v5.2.0 (SoftGenetics; https://www.softgenetics.com/products/mutation-surveyor/, accessed on 2 January 2026).

DNA was amplified by conventional polymerase chain reaction, followed by sequencing using the Applied BigDye^®^ Terminator v3.1 Cycle Sequencing Kit protocol (Applied Biosystems, Thermo Fisher Scientific, Waltham, MA, USA), and the Prism 3130XL sequencing apparatus (v3.1 Cycle Sequencing, Applied Biosystem, Foster City, CA, USA). Electropherograms were visualized in the CLC Main Workbench (Applied Biosystems, Thermo Fisher Scientific, Waltham, MA, USA).

### 4.3. Genetic Variants Pathogenicity Prediction

To assess the impact of the variant on splicing, we used Human Splice Finder version 3.0 (HSF 3.0) [41]. PolyPhen-2 was used to predict the potentially damaging effects of the variants on protein function [42]. We applied RNAz to evaluate the effects on RNA secondary structure and stability [43], lncRNASNP2 to predict their influence on lncRNA function and regulatory roles [44], and RPISeq to investigate the impact of variants on RNA–protein interactions [45]. We also predicted the protein’s three-dimensional structure using ColabFold v.1.6.1, an AlphaFold-based tool [46]. The predicted protein structures were visualized with PyMOL tool v.3.1.6.1 (https://www.pymol.org/).

To investigate the potential regulatory role of the long non-coding RNA *PDE11A-AS1*, the variants rs17400325 and rs776984134 were annotated using the Variant Effect Predictor (VEP) [47] to determine their genomic localization within the *PDE11A/PDE11A-AS1* locus.

Human genome reference and gene annotations (GRCh38) from Ensembl were used to retrieve all annotated transcripts of *PDE11A* and *PDE11A-AS1*. In total, 12 *PDE11A* and five *PDE11A-AS1* transcripts were selected for subsequent analyses. RNA–RNA interaction predictions between antisense *PDE11A-AS1* and sense *PDE11A* transcripts were performed using IntaRNA v.3.4.1 [48]. Pairwise analyses were conducted for all transcript combinations. Hybridization energies and predicted interaction regions were computed for each transcript pair.

RNA–protein interaction predictions involving *PDE11A-AS1* were performed using catRAPID omics v2.1 [49]. The reference transcript sequence NR_136171.1 was used as input in the interaction prediction module against the precompiled RNA-binding proteome, generating predicted interaction scores for RNA–protein pairs. Gene Ontology enrichment analysis was subsequently conducted using g:Profiler (https://biit.cs.ut.ee/gprofiler/gost, accessed on 4 February 2026) [50] on proteins with interaction propensity scores above 70 for *PDE11A-AS1*, thereby identifying overrepresented molecular functions, biological processes, and cellular components.

Domain annotation of the MSH6 protein was performed using the InterPro database (https://www.ebi.ac.uk/interpro/, accessed on 13 January 2026) to identify functional regions and map the p.Lys852 deletion to known protein domains. Three-dimensional structural models of both the wild-type and p.Lys852 variant proteins, encompassing residues 608–1082, were generated using the AlphaFold Protein Structure Database (AlphaFold server) [51]. Structural superposition and visualization were carried out in PyMOL (Schrödinger, LLC), and root mean square deviation (RMSD) values were calculated to assess global structural similarity between models. Local structural differences were evaluated by measuring distances between backbone atoms in the region surrounding the deletion. Per-residue confidence scores predicted by AlphaFold (pLDDT), encoded in the B-factor field of the models, were visualized using a color spectrum in PyMOL to assess differences in local structural confidence between the MSH6 wild-type variant and the p.Lys852 variant protein.

Gene-specific expression boxplots for *PDE11A*, *MSH6*, and *CTU2* were generated using VST-normalized RNA-seq data from TCGA-TGCT and GTEx testis samples obtained through the UCSC Xena platform. Differences in expression between tumor and normal samples were evaluated using the Wilcoxon rank-sum test, and visualizations were generated in R with the ggplot2 package.

The protein–protein interaction (PPI) network associated with PDE11A was constructed using the STRING database and visualized in Cytoscape v.3.9.1. To ensure high-confidence network inference, only interactions with a STRING confidence score ≥ 0.9 were retained. Proteins identified within the PDE11A interaction network were subsequently analyzed for GO biological process enrichment using Enrichr (https://maayanlab.cloud/Enrichr/, accessed on 18 May 2026). Enriched terms were ranked according to the combined score, which integrates enrichment significance and deviation from the expected rank. Biological processes with an adjusted *p*-value < 0.05 were considered significantly enriched. The most significantly enriched biological processes were visualized as a bar plot using the ggplot2 package v.4.0.3 in R v.4.5.1.

## 5. Conclusions

This family-based study identifies compound heterozygous *PDE11A* variants in monozygotic twins with testicular germ cell tumor and prioritizes *PDE11A* as a candidate predisposition locus. The splice-site allele rs776984134 is the strongest candidate variant, whereas rs17400325 may have context-dependent or modifier-level significance. Notably, rs17400325 also overlaps the *PDE11A-AS1* genomic region, suggesting a potential regulatory role for this transcript. In addition to the structural and functional impacts on *PDE11A*, the identified variants may also influence the regulation of the lncRNA *PDE11A-AS1,* which is transcribed at the same locus. Although the variant is predicted to affect the catalytic site of the PDE11A protein, it may also modulate PDE11A-AS1 activity, which could contribute to TGCT risk. Alterations affecting both protein structure and RNA-level regulation may contribute to *PDE11A* dysfunction. In this context, *PDE11A* emerges as a strong candidate gene for TGCT predisposition in this family, highlighting the importance of evaluating variants in overlapping sense–antisense genomic regions in hereditary cancer studies. Moreover, although the identified *MSH6* variant is currently classified as a VUS according to ACMG/ClinVar criteria, our findings support its potential relevance to the genetic background of TGCT. Similarly, the *CTU2* variant, previously reported as pathogenic in the homozygous state, may confer disease susceptibility in the heterozygous state, although its specific role in TGCT remains to be clarified. Our findings support the hypothesis that familial TGCT susceptibility may involve a complex multigenic architecture, in which variants affecting distinct biological pathways and regulatory mechanisms could contribute collectively to disease risk rather than resulting from a single highly penetrant genetic event.

## Figures and Tables

**Table 1:** Germline variants identified in twins in a family-based analysis.

Chr:Pos	Ref/Alt	Gene Name	Effect	gnomAD Variant Frequency	dbSNP154 RS ID	ACMG/ ClinVar	Inheritance	Sample Phorank Gene Score
chr1:3411594	G/T	*PRDM16*	Missense	7.52549 × 10^−6^	rs376891320	VUS/-	Paternal	0.971175
chr1:10640051	T/C	*CASZ1*	Missense	1.10103 × 10^−5^	rs768118953	VUS/VUS	Maternal	0.980593
chr1:37616655	C/T	*RSPO1*	Missense	0.0015954	rs201499112	VUS/VUS	Paternal	1.19872
chr1:110064736	T/C	*ALX3*	Missense	-	-	VUS/-	Paternal	0.872169
chr1:115838282	C/T	*NHLH2*	Missense	0.000109311	rs774831438	VUS/VUS	Maternal	0.934577
chr1:228317556	G/A	*OBSCN*	Missense	0.000466623	rs201223554	VUS/-	Paternal	0.362793
chr2:27239469	C/A	*CAD*	Missense	0.000786412	rs138840581	VUS/VUS	Maternal	0.359434
chr2:47800537	AAG/-	*MSH6*	Missense	2.46364 × 10^−5^	rs587782858	VUS/VUS	Maternal	2.21997
chr2:177680905	T/C	*PDE11A,* *PDE11A-AS1*	LoF	7.26062 × 10^−6^	rs776984134	VUS/-	Paternal	2.73167
chr2:177701185	T/C	*PDE11A,* *PDE11A-AS1*	Missense	0.0369668	rs17400325	Benign/-	Maternal	2.73167
chr3:10049425	G/A	*FANCD2*	Missense	5.47719 × 10^−6^	rs761454597	VUS/VUS	Paternal	0.952159
chr3:43080422	T/C	*POMGNT2*	Missense	0.000128603	rs200535361	VUS/Conflicting	Paternal	0.744607
chr3:47123032	T/C	*SETD2*	Missense	1.43665 × 10^−5^	rs756943490	VUS/VUS	Paternal	0.636365
chr4:87663280	T/C	*DMP1*	Missense	7.3877 × 10^−5^	rs146762807	VUS/VUS	Maternal	0.327186
chr5:475021	C/T	*SLC9A3,* *SLC9A3-AS1*	Missense	6.84861 × 10^−7^	rs747804305	VUS/-	Maternal	0.726474
chr6:44253579	G/A	*HSP90AB1,* *POLR1C*	Missense	3.34341 × 10^−5^	rs76772157	VUS/-	Maternal	0.915541
chr6:157190108	A/G	*ARID1B*	Missense	3.83073 × 10^−5^	rs138784762	VUS/Conflicting	Maternal	1.05003
chr7:33014776	T/C	*NT5C3A*	Missense	0.00426816	rs79747830	VUS/-	Maternal	0.368528
chr10:47325295	G/T	*GDF2*	Missense	8.89278 × 10^−6^	rs782573337	VUS/VUS	Maternal	0.908666
chr10:58813920	G/A	*BICC1*	Missense	1.36825 × 10^−6^	-	VUS/-	Maternal	1.02452
chr11:65720155	T/C	*RNASEH2C*	Missense	4.10429 × 10^−5^	rs145783593	VUS/VUS	Paternal	0.896403
chr11:66055305	G/A	*SF3B2*	Missense	1.36995 × 10^−5^	rs146650893	VUS/-	Maternal	0.339226
chr11:119025041	T/C	*SLC37A4*	Missense	4.65216 × 10^−5^	rs782480303	VUS/VUS	Paternal	1.07133
chr16:10923254	G/A	*CIITA*	Missense	0.000361999	rs138790505	VUS/VUS	Maternal	0.322608
chr16:67647564	G/A	*CARMIL2*	Missense	0.000105554	rs371235689	VUS/VUS	Maternal	0.882961
chr16:67654664	A/C	*CARMIL2*	Missense	0.00158804	rs200875393	VUS/Conflicting	Maternal	0.882961
chr16:88709982	T/C	*CTU2*	Missense	0.00110151	rs147948789	LP/P	Maternal	0.712685
chr18:9122613	T/C	*NDUFV2,* *NDUFV2-AS1*	Missense	0.000182714	rs72935225	VUS/-	Paternal	0.332309
chr18:23882016	C/G	*LAMA3*	Missense	9.58029 × 10^−6^	rs761924134	VUS/-	Maternal	0.90049
chr18:70030953	A/G	*RTTN*	Missense	2.66918 × 10^−5^	rs369861224	VUS/VUS	Paternal	0.751557
chr20:13580942	C/A	*TASP1*	Missense	1.16386 × 10^−5^	rs749135048	VUS/-	Maternal	0.735785
chr20:62314419	C/T	*LAMA5*	Missense	0.000194363	rs200140507	VUS/VUS	Maternal	0.682388
chr21:45510107	G/A	*COL18A1*	Missense	0.00232029	rs200484625	VUS/Conflicting	Maternal	0.344417
chr22:35413981	A/G	*MCM5*	Missense	1.50818 × 10^−5^	rs542801531	VUS/VUS	Maternal	0.727837

Chr:Pos: Chromosomal Position; Ref/Alt: Reference/Alternate; LoF: Loss of Function; P: Paternal.

## Data Availability

The datasets generated in this study have been deposited at the Sequence Read Archive (SRA, https://www.ncbi.nlm.nih.gov/sra, accessed on 8 April 2026) under accession number PRJNA1450532.

## Supplementary Materials

The following supporting information can be downloaded at: https://www.mdpi.com/article/10.3390/ijms27125261/s1.
