# Supplementary material for: Family-Based Study Reveals PDE11A/PDE11A-AS1 Variants in Testicular Germ Cell Tumor Predisposition
Source: Int J Mol Sci. 2026 Jun 10;27(12):5261. doi: 10.3390/ijms27125261 (PMC13300246; doi:10.3390/ijms27125261)
Supplement: Supplementary file 1 [file ijms-27-05261-s001.zip › Supplementary Table S2 and Figures S1-S7.pdf]

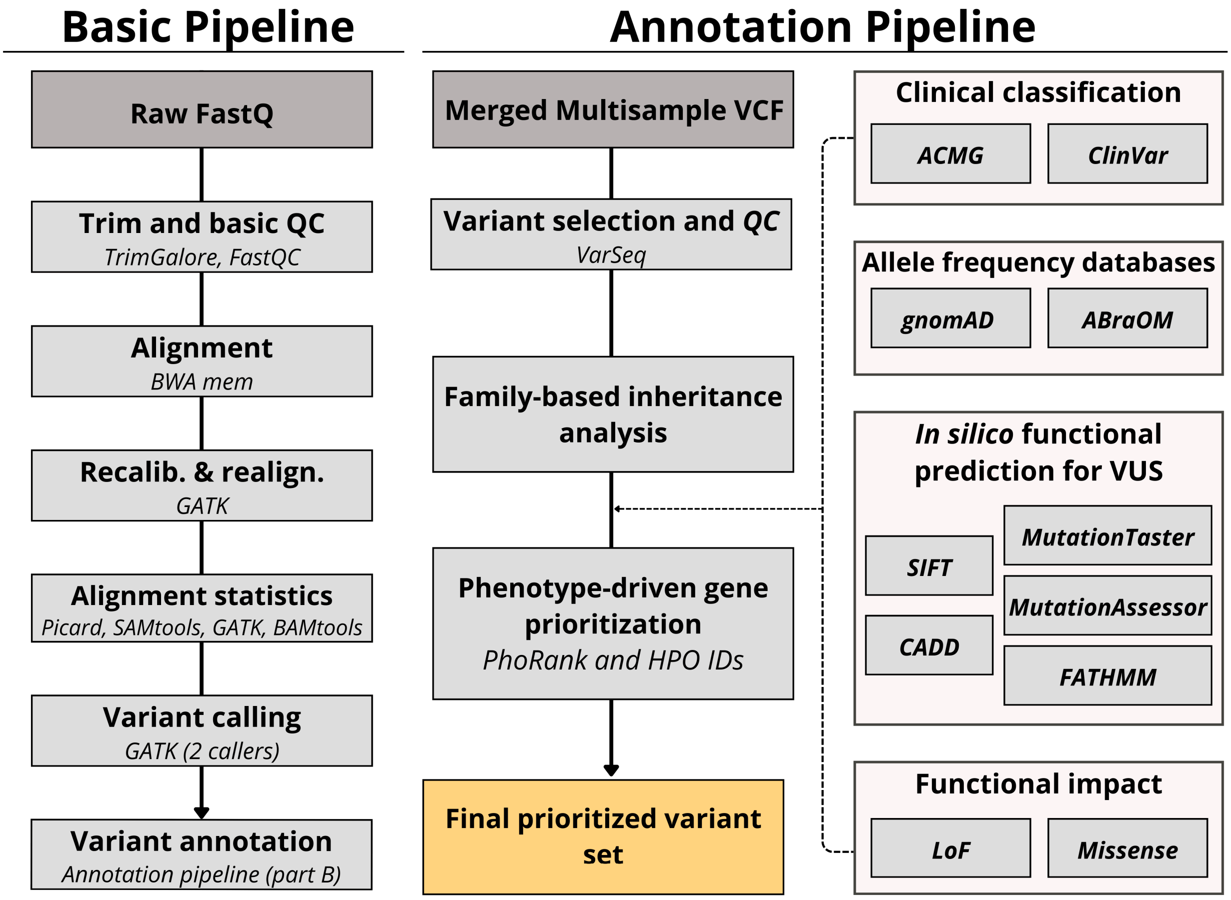

Figure S1. Whole-exome sequencing (WES) based on the trio analysis workflow. The Basic Pipeline encompasses raw read processing, quality control, alignment, recalibration, and realignment, followed by variant calling and initial annotation. The Annotation Pipeline integrates multisample VCF processing with variant filtering, inheritance-based prioritization, phenotype-guided gene selection using Human Phenotype Ontology (HPO) terms, clinical classification, allele frequency evaluation, and *in silico* functional assessment. This workflow generates a final, prioritized set of variants for downstream interpretation.

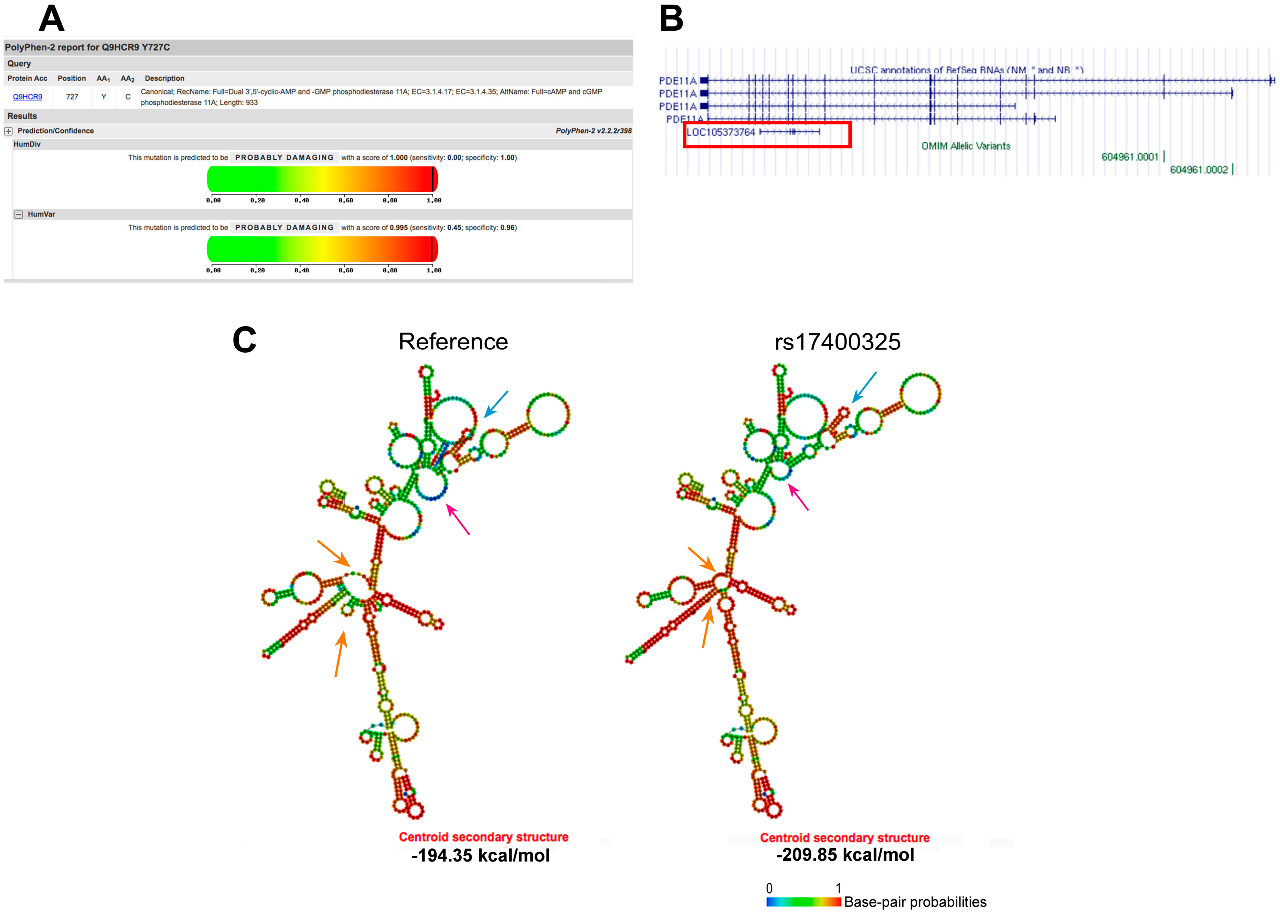

Figure S2. Predictions of the pathogenicity of *PDE11A* variants. (**A**) PolyPhen-2 analysis of PDE11A p.Tyr727Cys variant in (**B**). Localization of the lncRNA *PDE11A-AS1* (**C**) RNA secondary structure analysis of *PDE11A-AS1* wild type and mutant sequence containing the rs17400325 variant. Colored arrows indicate the differences.

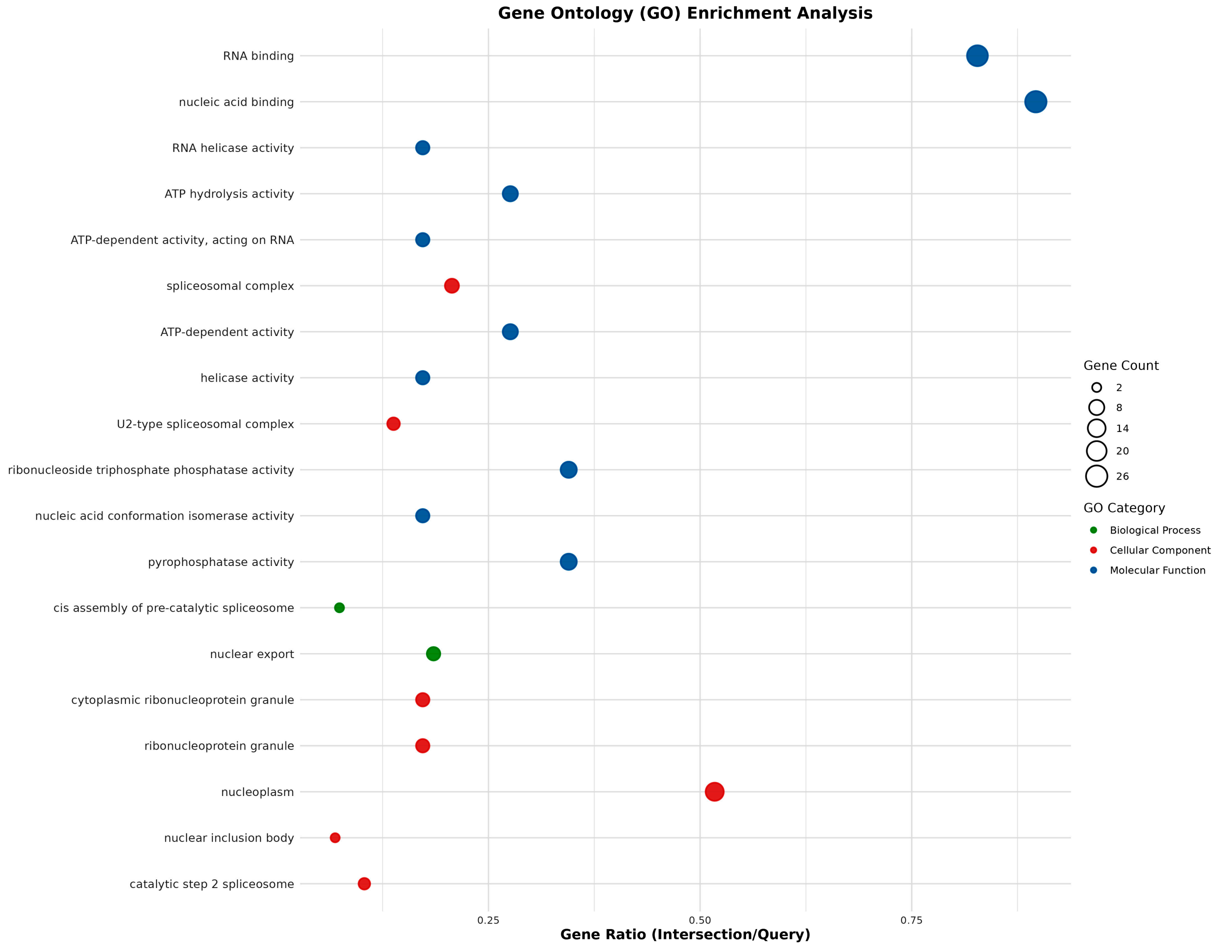

Figure S3. Gene Ontology enrichment analysis of high-confidence *PDE11A-AS1* protein interactors (interaction propensity > 70). The top enriched Molecular Function, Biological Process, and Cellular Component terms identified with g:Profiler, with dot size indicating the number of proteins associated with each term. The x-axis represents the gene ratio.

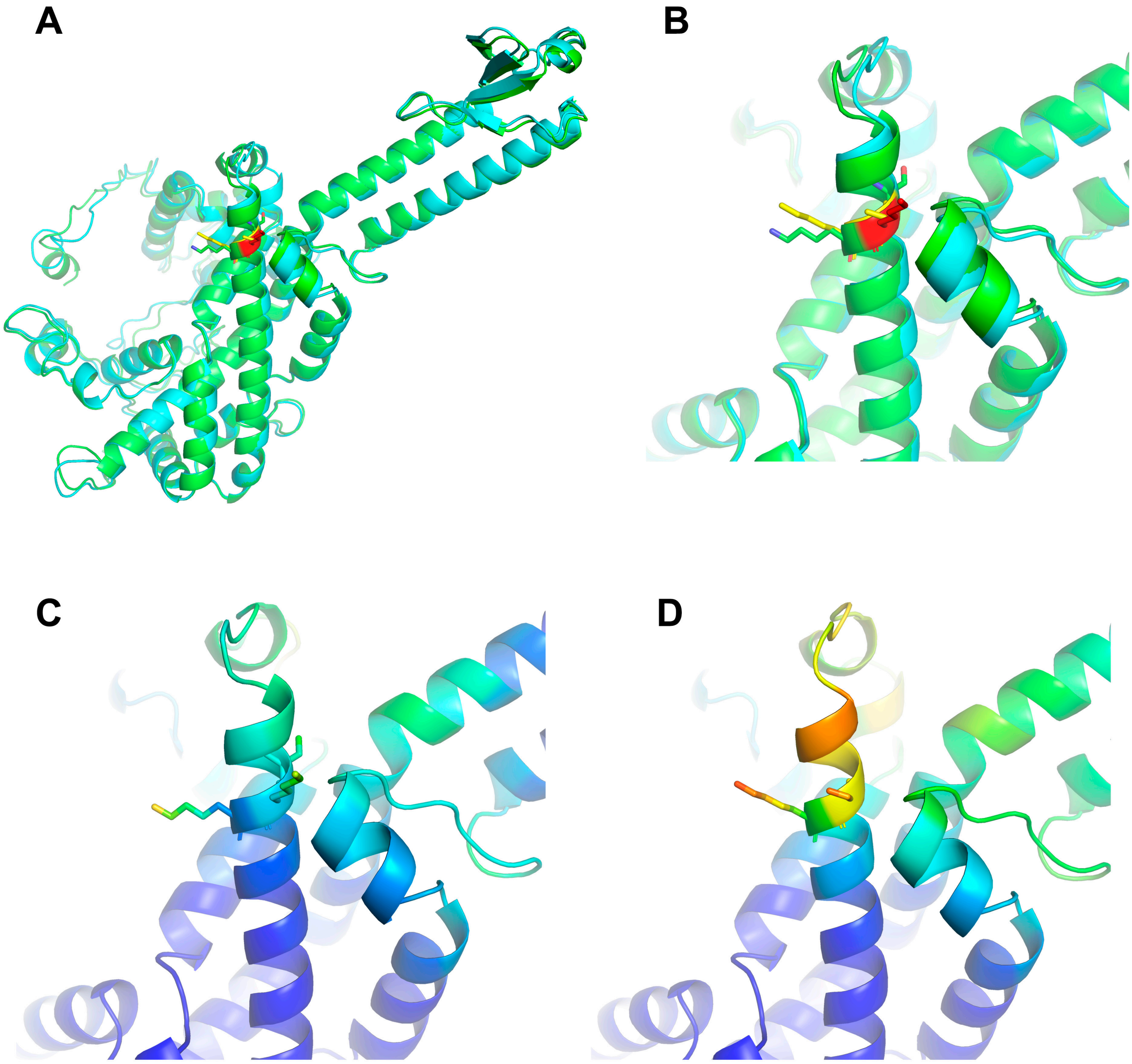

Figure S4. AlphaFold-based structural analysis of MSH6 p.Lys852 in-frame deletion (**A**) Global alignment of AlphaFold-predicted structures spanning residues 608–1082 of wild-type *MSH6* (green) and the rs587782858 variant (cyan), showing preservation of the overall protein fold. (**B**) Zoomed view of the aligned structures at the Lys852 region, highlighting the local structural differences associated with the in-frame deletion. The lysine residue in the wild-type protein is shown in red, while the corresponding region in the rs587782858 variant is shown in yellow. (**C**) pLDDT confidence score mapping of the wild-type *MSH6* model. (**D**) pLDDT confidence score mapping of the rs587782858 variant model, showing reduced local structural confidence at the deletion site.

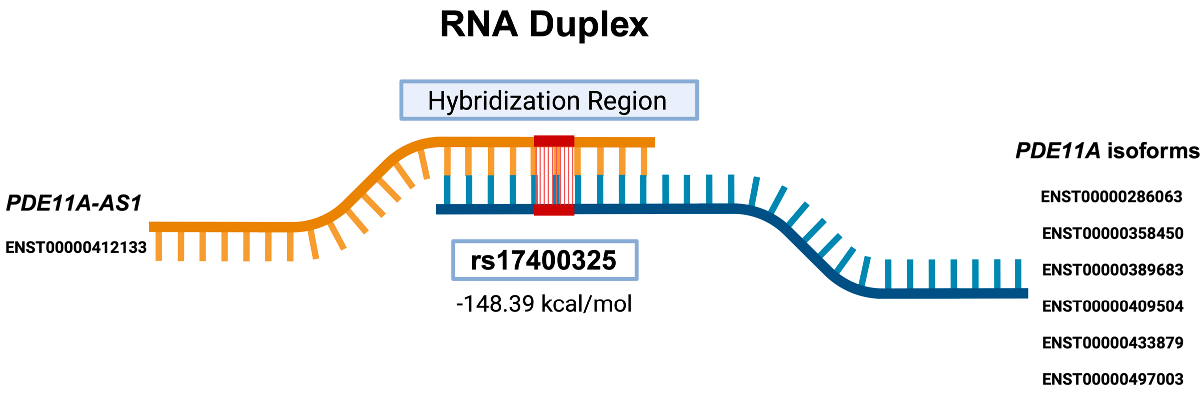

Figure S5. Among the six interactions involving the *PDE11A-AS1* transcript ENST00000412133 and the subset of *PDE11A* transcripts, rs17400325 is located within the predicted RNA–RNA hybridization region in both antisense and sense transcripts. These interactions consistently involve the same *PDE11A-AS1* segment (nucleotides 133–281), paired with distinct regions across the six *PDE11A* isoforms.

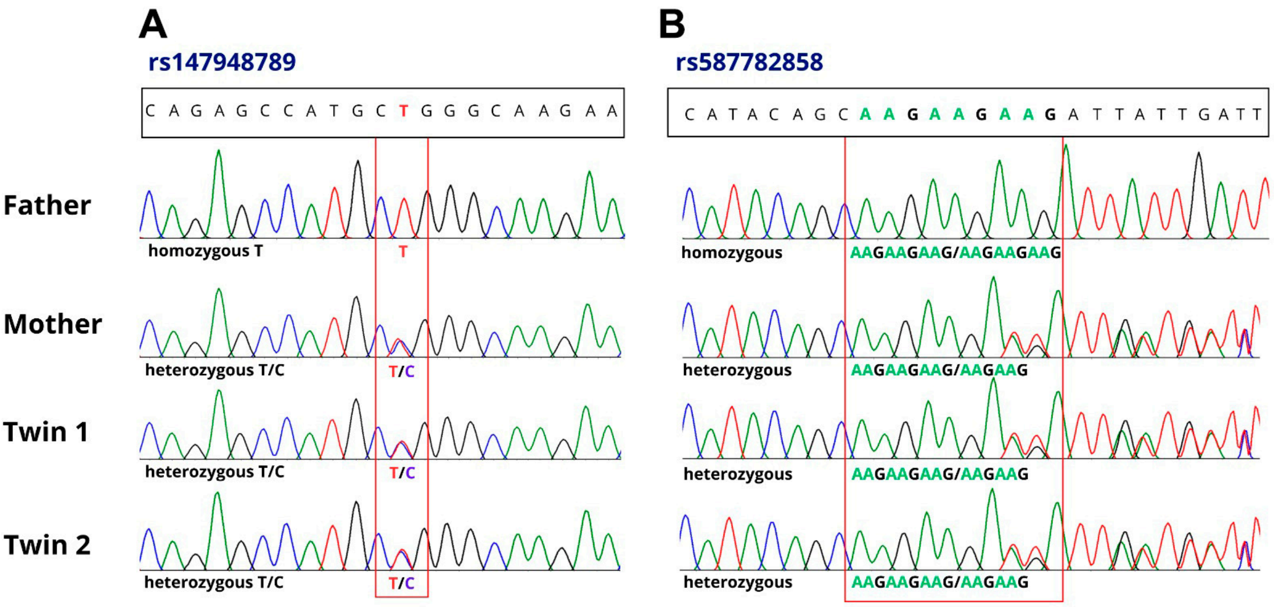

Figure S6. Sanger sequencing chromatograms showing the identified variants. (**A**) Variant rs147948789 in *CTU2* (c.188T>C; p.Leu63Pro). The mother and both twins (Twin 1 and Twin 2) are heterozygous (T/C), whereas the father is homozygous for the wild-type allele (T/T). (**B**) Variant rs587782858 in *MSH6* (c.2643_2645del; p.Lys852del). The mother and both twins are heterozygous for the *MSH6* deletion, whereas the father is homozygous for the wild-type allele. The variant positions are highlighted by red boxes. In panel A, the T nucleotide is shown in red and the C nucleotide in blue. In panel B, the deleted nucleotides are highlighted in green.

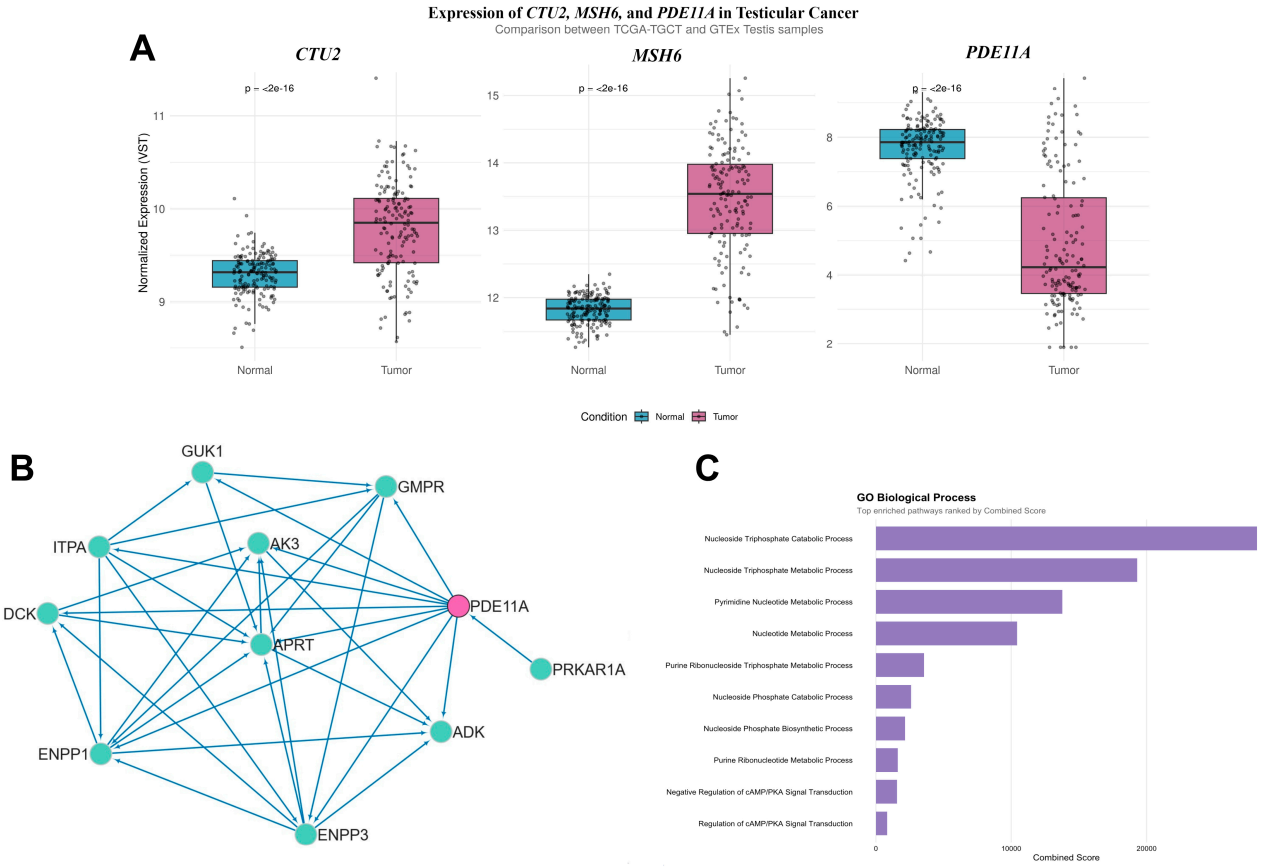

Figure S7. External transcriptomic datasets and systems biology analysis support *PDE11A* as a putative TGCT susceptibility locus. (**A**) Expression patterns of *CTU2*, *MSH6*, and *PDE11A* in normal testicular tissues and TGCT samples based on GTEx and TCGA datasets. *PDE11A* showed reduced expression in TGCT samples, whereas *MSH6* and *CTU2* exhibited increased expression in tumor tissues compared to normal testis. (**B**) Protein–protein interaction (PPI) network generated in Cytoscape using STRING with a high-confidence interaction score (0.9). The network illustrates the functional associations between *PDE11A* and its interacting genes involved in nucleotide metabolism and signaling pathways. (**C**) Gene Ontology (GO) Biological Process enrichment analysis of genes from the interaction network. Bar plot representation of the top enriched biological processes ranked according to combined score, highlighting pathways related to nucleoside triphosphate metabolism, nucleotide metabolic processes, and regulation of cAMP/PKA signal transduction.

Table S2. IntaRNA-Predicted RNA–RNA Interaction Energies Between *PDE11A* and *PDE11A-AS1* Transcripts.

Antisense transcript ID	Antisense interaction start	Antisense interaction end	Sense transcript ID	Sense interaction start	Sense interaction end	Predicted RNA–RNA interaction energy (kcal/mol)
ENST00000412133*	133	281	ENST00000286063	2472	2619	-148.39
ENST00000450227	386	450	ENST00000286063	2362	2425	-18.83
ENST00000653062	1338	1486	ENST00000286063	4246	4380	-22.6
ENST00000815670	68	131	ENST00000286063	3284	3351	-20.26
ENST00000815671	31	96	ENST00000286063	218	281	-32.72
ENST00000412133*	133	281	ENST00000358450	1481	1628	-148.39
ENST00000450227	386	450	ENST00000358450	1371	1434	-18.83

ENST00000653062	1338	1486	ENST00000358450	3255	3389	-22.6
ENST00000815670	68	131	ENST00000358450	2293	2360	-20.26
ENST00000815671	41	90	ENST00000358450	2302	2357	-23.61
ENST00000412133*	133	281	ENST00000389683	950	1097	-148.39
ENST00000450227	386	450	ENST00000389683	840	903	-18.83
ENST00000653062	14	86	ENST00000389683	706	783	-18.03
ENST00000815670	614	678	ENST00000389683	840	903	-18.87
ENST00000815671	494	525	ENST00000389683	1259	1294	-15.69
ENST00000412133*	133	281	ENST00000409504	1149	1296	-148.39
ENST00000450227	386	450	ENST00000409504	1039	1102	-18.83
ENST00000653062	1007	1071	ENST00000409504	1889	1949	-20.41
ENST00000815670	68	131	ENST00000409504	1884	1951	-20.31
ENST00000815671	41	90	ENST00000409504	1893	1948	-25.77
ENST00000412133	501	533	ENST00000427127	24	69	-18.35
ENST00000450227	305	337	ENST00000427127	24	69	-18.35
ENST00000653062	731	763	ENST00000427127	24	69	-18.59
ENST00000815670	528	560	ENST00000427127	24	69	-18.34
ENST00000815671	491	523	ENST00000427127	24	69	-18.59
ENST00000412133*	133	281	ENST00000433879	955	1102	-148.39
ENST00000450227	386	450	ENST00000433879	845	908	-18.83
ENST00000653062	14	86	ENST00000433879	711	788	-18.03
ENST00000815670	614	678	ENST00000433879	845	908	-18.87
ENST00000815671	494	525	ENST00000433879	1264	1299	-17.12
ENST00000412133	143	164	ENST00000436700	815	837	-15.84
ENST00000450227	346	421	ENST00000436700	111	168	-13.97
ENST00000653062	36	99	ENST00000436700	551	618	-19.95
ENST00000815670	68	131	ENST00000436700	551	618	-20.26
ENST00000815671	41	90	ENST00000436700	560	615	-23.61
ENST00000412133	194	226	ENST00000466790	341	370	-19.56
ENST00000450227	308	339	ENST00000466790	340	375	-15.3

ENST00000653062	734	765	ENST00000466790	340	375	-15.74
ENST00000815670	531	570	ENST00000466790	338	375	-15.32
ENST00000815671	494	525	ENST00000466790	340	375	-15.74
ENST00000412133	194	226	ENST00000478646	53	82	-15.4
ENST00000450227	221	299	ENST00000478646	82	153	-14.19
ENST00000653062	1032	1071	ENST00000478646	485	523	-19.43
ENST00000815670	68	131	ENST00000478646	480	547	-17.93
ENST00000815671	41	90	ENST00000478646	489	544	-21.44
ENST00000412133	196	226	ENST00000488399	196	223	-20.21
ENST00000450227	346	421	ENST00000488399	258	315	-14.59
ENST00000653062	235	268	ENST00000488399	491	523	-18.34
ENST00000815670	56	143	ENST00000488399	226	309	-18.77
ENST00000815671	32	59	ENST00000488399	336	369	-15.54
ENST00000412133	410	439	ENST00000492761	619	647	-19.66
ENST00000450227	214	243	ENST00000492761	619	647	-19.66
ENST00000653062	640	669	ENST00000492761	619	647	-19.67
ENST00000815670	437	466	ENST00000492761	619	647	-19.67
ENST00000815671	400	429	ENST00000492761	619	647	-19.67
ENST00000412133*	133	281	ENST00000497003	1174	1321	-148.39
ENST00000450227	359	396	ENST00000497003	2387	2437	-20.58
ENST00000653062	1	65	ENST00000497003	1	60	-26.46
ENST00000815670	48	178	ENST00000497003	2336	2472	-24.76
ENST00000815671	65	139	ENST00000497003	2380	2452	-21.24

* *PDE11A* transcripts with highly favorable interactions with *PDE11A-AS1* (ENST00000412133), as indicated by strongly negative IntaRNA-predicted hybridization energies (−148.39 kcal/mol).
